# Molecular Diagnosis of Periprosthetic Joint Infection by Quantitative RT-PCR of Bacterial 16S Ribosomal RNA

**DOI:** 10.1155/2013/950548

**Published:** 2013-12-17

**Authors:** Mel S. Lee, Wen-Hsin Chang, Su-Chin Chen, Pang-Hsin Hsieh, Hsin-Nung Shih, Steve W. N. Ueng, Gwo-Bin Lee

**Affiliations:** ^1^Department of Orthopaedic Surgery, Chia-Yi Chang Gung Memorial Hospital, 6 West Sec., Chiapu Road, Putzu City, Chiayi County 613, Taiwan; ^2^Department of Orthopaedic Surgery, Linkou Chang Gung Memorial Hospital, Putzu City, Chiayi County 613, Taiwan; ^3^College of Medicine, Chang Gung University, Putzu City, Chiayi County 613, Taiwan; ^4^Department of Power Mechanical Engineering, National Tsing Hua University, No. 101, Section 2, Kuang-Fu Road, Hsinchu 30013, Taiwan; ^5^Institute of NanoEngineering and Microsystems, National Tsing Hua University, Hsinchu 30013, Taiwan

## Abstract

The diagnosis of periprosthetic joint infection is sometimes straightforward with purulent discharge from the fistula tract communicating to the joint prosthesis. However it is often difficult to differentiate septic from aseptic loosening of prosthesis because of the high culture-negative rates in conventional microbiologic culture. This study used quantitative reverse transcription polymerase chain reaction (RT-qPCR) to amplify bacterial 16S ribosomal RNA in vitro and in 11 clinical samples. The in vitro analysis demonstrated that the RT-qPCR method was highly sensitive with the detection limit of bacterial 16S rRNA being 0.148 pg/**μ**l. Clinical specimens were analyzed using the same protocol. The RT-qPCR was positive for bacterial detection in 8 culture-positive cases (including aerobic, anaerobic, and mycobacteria) and 2 culture-negative cases. It was negative in one case that the final diagnosis was confirmed without infection. The molecular diagnosis of bacterial infection using RT-qPCR to detect bacterial 16S rRNA around a prosthesis correlated well with the clinical findings. Based on the promising clinical results, we were attempting to differentiate bacterial species or drug-resistant strains by using species-specific primers and to detect the persistence of bacteria during the interim period before the second stage reimplantation in a larger scale of clinical subjects.

## 1. Introduction

Periprosthetic infection (PJI) is difficult to treat and sometimes to diagnose. To differentiation septic from aseptic loosening is often challenging because the PJI might be partially suppressed by antibiotics before the loosening of the prosthesis. The utilization of medical resources in treating PJI is 2.8 times higher than that associated with revision surgery because of aseptic loosening [1, 2]. Definite diagnosis of PJI before revision surgery is therefore important because it reduces perioperative risks and medical costs. The American Musculoskeletal Infection Society has recently published new diagnostic criteria for a definite PJI which consists 1 of the 2 major criteria (sinus tract communicating with prosthesis or at least 2 positive tissue culture results) or 4 of the 6 minor criteria [3]. For probable or possible PJI, a consensus has not yet been reached. Because of the high incidence of culture-negative rates in clinical practice, surgeons need to make the decision based on every evidence to determine whether a revision surgery could be performed or an extended period of antibiotics therapy should be commenced.

For those patients with confirmed PJI, a two-stage reimplantation protocol that consists of extensive debridement at the first stage followed by delayed reimplantation is currently the standard of care in many hospitals with the success rate being between 82% and 95% [4–8]. The timing of reimplantation arthroplasty depends on the complete eradication of infection to avoid devastating complications [9]. Diagnostic methods such as the serum CRP, interleukin-6, culture of joint aspirates, bone scans, frozen sections, and other molecular markers are the most commonly used surrogate parameters to determine the complete eradication of infection [10–17]. However, these tests have limitations such as being time consuming or nonspecific for the diagnosis of infection persistence. Previous studies used bacterial ribosomal RNA (rRNA) as a target for the diagnosis of infection [18]. The rRNAs are highly conserved among bacterial species, abundant in amount, and not present in human. The rRNA can be amplified by RT-PCR. Currently the detection limit of RT-PCR for bacterial rRNA is highly sensitive and avoids the high false-positive rates of amplifying the bacterial DNA [10, 18–21]. It can be served as a cell viability marker to differentiate dead organism from active infection [18, 22]. The purpose of this study was to test the feasibility of using RT-qPCR of bacterial 16S rRNA in the detection of PJI by in vitro and clinical specimens.

## 2. Materials and Methods

### 2.1. In Vitro rRNA Detection Limits

Total RNA was isolated from samples for the purposes of detecting rRNA of the assay. The same protocol was used to evaluate clinical samples. Enzymatic bacterial lysis was performed to ensure release of all intracellular RNA species in the samples. One milliliter of each sample was pipetted 2 volumes of RNAprotect Bacteria Reagent (QIAGEN, Valencia, California). The RNeasy Mini Kit (QIAGEN, Valencia, California) was used for column purification of total RNA. Poly(A) RNA (20 ng/5 mL) was used as a carrier species and was added to the specimen before using the RNeasy column to improve RNA yield with dilute samples. DNA contamination was eliminated by means of on-column DNase digestion prior to elution of total RNA from the column with 120 mL of RNase-free water. A 5-mL aliquot of total bacterial RNA was analyzed by the iScript one-step RT-PCR Kit with SYBR Green on an iCycler Thermal Cycler (Bio-Rad, Hercules, California) using universal primer pairs of bacterial 16S rRNA (forward 5′-attagataccctggtagtccacgcc-3′; reverse 5′-cgtcatccccaccttcctcc-3′). The cycling conditions were 50° for 10 minutes and 95° for 5 minutes, followed by 35 cycles of 95° for 10 seconds and 62° for 30 seconds. Limited dilution of standard strains of *E. coli* (dH10*β*) was used to analyze the detection limits of the assay.

### 2.2. Clinical Specimen Analysis

Joint fluids from patients who were suspected to have PJI were collected during operation. With informed consent and IRB approval (IRB no. 101-3480A3), demographic data, medical history, laboratory data, and culture results were recorded. The joint fluid was aliquot and subjected to enzymatic bacterial lysis after treating it with RNAprotect Bacteria Reagent (QIAGEN, Valencia, California) as described above. All RT-qPCR protocol was identical to the in vitro analysis except one of the samples was spiked with the standard strain of *E. coli* to serve as a positive control.

## 3. Results

### 3.1. Detection Limit

**Figure 1:**
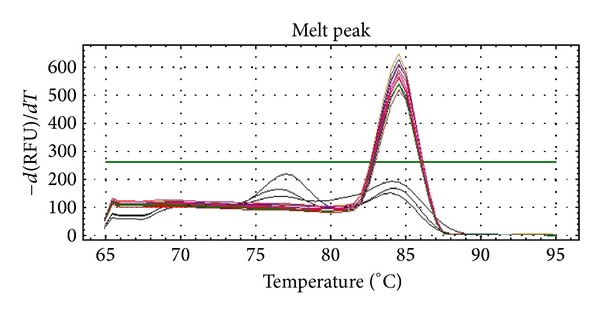
Melting temperature of all amplicons with serial dilution of total RNA.

**Figure 2:**
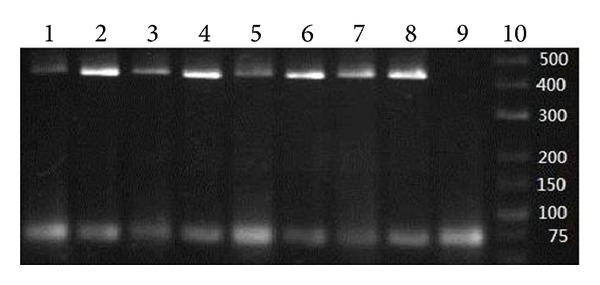
Gel electrophoresis of clinical specimens. Lanes 1, 3, 5, and 7: clinical specimen. Lanes 2, 4, 6, and 8: spiked specimen loaded with RNA of the standard strain of *E. coli* as positive control. Lane 9: negative control. Lane 10: DNA ladder.

After an overnight culture of the standard strain of *E. coli*, an aliquot of 5 mL culture medium was subjected to total RNA extraction. The average yield of RNA was 1.48 ng/*μ*L. RT-qPCR was performed by a serial dilution of tenfold of the total RNA and analyzed on the iCycler Thermal Cycler (Bio-Rad, Hercules, California). It was found at the detection limit of the system which was at the total RNA concentration of 0.148 pg/*μ*L. The melting temperature of all amplicons with serial dilution was similar between groups (Figure 1). Results were further checked with gel electrophoresis and showed consistent results as the RT-qPCR (Figure 2).

### 3.2. Clinical Specimens

In the study period, there were 11 patients referred for the diagnosis and treatment of PJI. Of the 11 patients, 10 were definite PJI based on the clinical presentations, laboratory data, and pathologic diagnosis (Table 1). One indeterminate case (case 11) was a staged reimplantation THA case and experienced swelling around the joint. Exploration of the hip joint revealed clear joint fluid and no evidence of infection by pathologic diagnosis. The culture was negative and the RT-qPCR result was negative for infection.

In the 10 confirmed PJI cases, the RT-qPCR results were all positive for infection. Among them, the culture result was no growth of bacteria in 2 cases. One case (case 4) had multiple organisms infection associated with a THA. One case (case 5) had mycobacterium infection. One case (case 6) had anaerobic bacteria infection. The RT-qPCR using the universal primers for 16S rRNA detection could identify bacterial infection including aerobic, anaerobic, and mycobacteria and those 2 culture-negative cases. In the 11 clinical specimens, the RT-qPCR test was found to be highly accurate in the diagnosis of PJI.

## 4. Discussion

PJI is a devastating complication for the patient and the health care providers. Its incidence is between 1% and 3% in primary and 4% and 6% in revision total joint arthroplasties [1, 2]. The diagnosis can be straightforward with purulent discharge from the joint but may also be confusing in indeterminate cases. Often infection leads to multiple operations, prolonged use of antibiotics, extensive utilization of medical resources, and substantial social, economic, or even psychological impacts on the patients, family, hospitals, physicians, and payers [2]. An accurate diagnosis of PJI remains a challenging clinical problem and is essential for the success of treatment.

For a two-stage protocol, the existence of living bacteria in the joint is contraindicated for the reimplantation procedure. Usually the decision is made by assessing the wound condition, checking ESR and CRP levels, joint aspiration for analysis and culture, intraoperative frozen sections, or with the help of radioisotope scintigraphy [14, 15, 23]. Unfortunately, these tests are limited in the diagnostic power. A false negative result might lead to repeated surgery and devastating complications.

Bacterial ribosomal RNA (rRNA) has been used as a target for the diagnosis of infection [18]. The 16S rRNA is unique in bacterial species and is highly sensitive as a cell viability marker to differentiate dead organism from active infection [18, 22]. In a study of 64 patients who were suspected of having infection around the total knee arthroplasty, the overall accuracy by using RT-qPCR to detect PJI was 94% [18]. In this study, we were able to detect the 16S rRNA at the picogram levels in vitro. In the clinical specimens, we successfully identified bacterial infection in 10 definite PJI cases including those 2 culture-negative cases. Although the results were still preliminary, the RT-qPCR method using universal primer pairs targeting the 16S rRNA was found to be feasible to detect common bacterial (both aerobic and anaerobic) and mycobacterial infection. The result could help the clinical decision making especially in those cases with negative bacterial culture results. Jacovides et al. used a PCR-based mass spectrometry in 87 arthroplasty procedures and detected bacterial infection in 4 of 5 culture-negative cases and 50 of 57 presumably noninfectious cases [24]. They concluded that the molecular diagnosis of PJI could not only be effective at detecting organisms in culture-negative cases but also identify many of the revision cases that may have subclinical infection components.

In this study, we used universal primers for the 16S rRNA detection. Although the bacterial 16S rRNA is highly conserved, it does mark evolutionary distance and relatedness of organisms [25]. Universal primers that are complimentary to the conserved regions of the 16S rRNA gene could result in some variations in the end products detected by quantitative PCR. However it is difficult to differentiate bacterial species by using the universal primers. We could only differentiate the presence or absence of bacterial infection in our clinical specimens in this study. The choice of antibiotics or the drug-resistant strain detection could not be attained by the current method. These limitations could potentially be addressed by using species-specific primers or targeting on the drug-resistant genes [26, 27].

In conclusion, we found the molecular diagnosis of bacterial infection using RT-qPCR to detect bacterial 16S rRNA which was highly accurate in the diagnosis of PJI. Further studies to detect the persistence of bacteria during the interim period before the second stage reimplantation and to differentiate bacterial species or drug-resistant strains should be done to improve the diagnosis and treatment of the PJI.

## Figures and Tables

**Table 1:** Clinical data on patients with suspected infection status.

Case	Age	Sex	Diagnosis of PJI	ESR (mm/hr)*	CRP (mg/L)	Fistula	Culture^†^	qPCR result
1	46	F	THA infection	109	15.78	Present	NG	Positive
2	79	M	TKA infection	55	194.49	Present	*Pseudomonas aeruginosa *	Positive
3	68	M	THA infection	48	41.66	Present	NG	Positive
4	48	F	THA infection	NA	76.76	Present	CONS, Staph epi, and MRSA	Positive
5	63	M	TKA infection	124	51.54	Absent	M. chelonae	Positive
6	66	M	THA infection	73	96.76	Absent	*Peptostreptococcus *	Positive
7	73	F	TKA infection	74	18	Absent	*Staphylococcus aureus *	Positive
8	70	F	TKA infection	NA	273.83	Present	MRSA	Positive
9	63	M	THA infection	64	104.76	Absent	MSSA	Positive
10	70	F	Revision of TKA infection	NA	111.01	Absent	MRSA	Positive
11	32	M	Reactive synovitis	NA	12.39	Absent	NG	Negative

*NA: not available.

^†^NG: no growth; CONS: coagulase negative *staphylococcus*; Staph epi: *Staphylococcus  epidermidis*; MRSA: methicillin-resistant *Staphylococcus  aureus*; M. chelonae: *Mycobacterium  chelonae*.
